# Flexible Edge for Rubber Plates

**Published:** 1871-02

**Authors:** S. J. McDougall


### ARTICLE III.

### *Flexible Edge for Rubber Plates.*

By Dr. S. J. McDougall.

Have soft rubber rolled in sheets long enough to extend around the entire plate. Make your calculations on the model how far up and back you wish the plate to extend. Cut a strip of the flexible or soft rubber the desired width, run it around on the model and stick it there (rubber dissolved in chloroform answers a good purpose). Of course the upper and posterior portion of the flexible edge forms the upper and posterior portion of the plate.

Now with the trial plate on the model, wax up to, or a little above the lower edge of the flexible strip. When putting the case in the flask for packing, imbed the flexible strip in plaster so that, when the halves of the flask are separated for packing, the flexible strip will remain on the model. Pack and vulcanize.

The great objection to this kind of work is, it will last only from six to eighteen months.

A day or two since I had a letter from Dr. Dudley, of Salem, saying he had used and published it some years ago, before a patent was obtained.

